# Narrative Review: Low-Dose Ketamine for Pain Management

**DOI:** 10.3390/jcm12093256

**Published:** 2023-05-02

**Authors:** Alessandro Riccardi, Mario Guarino, Sossio Serra, Michele Domenico Spampinato, Simone Vanni, Dana Shiffer, Antonio Voza, Andrea Fabbri, Fabio De Iaco

**Affiliations:** 1 Emergency Department, Ospedale di Imperia, 18100 Imperia, Italy; dottriccardi@gmail.com; 2 Emergency Department, Centro Traumatologico Ortopedico, Azienda Ospedaliera di Rilievo Nazionale dei Colli, 80131 Napoli, Italy; marioguarino63@gmail.com; 3 Emergency Department, Maurizio Bufalini Hospital, 47522 Cesena, Italy; sossio.serra@gmail.com; 4 Department of Translational Medicine and for Romagna, University of Ferrara, 44124 Ferrara, Italy; 5 Dipartimento Emergenza e Area Critica, Azienda USL Toscana Centro Struttura Complessa di Medicina d’Urgenza, 50053 Empoli, Italy; simonevanni68@gmail.com; 6 Emergency Department, Humanitas University, Via Rita Levi Montalcini 4, 20089 Milan, Italy; dana.shiffer@humanitas.it; 7 Emergency Department, IRCCS Humanitas Research Hospital, 20089 Milan, Italy; antonio.voza@humanitas.it; 8 Emergency Department, AUSL Romagna, Presidio Ospedaliero Morgagni-Pierantoni, 47121 Forlì, Italy; andrea.fabbri@auslromagna.it; 9 Emergency Department, Ospedale Maria Vittoria, 10144 Turin, Italy; dr.fabio.deiaco@gmail.com

**Keywords:** ketamine, pain, emergency departments

## Abstract

Pain is the leading cause of medical consultations and occurs in 50–70% of emergency department visits. To date, several drugs have been used to manage pain. The clinical use of ketamine began in the 1960s and it immediately emerged as a manageable and safe drug for sedation and anesthesia. The analgesic properties of this drug were first reported shortly after its use; however, its psychomimetic effects have limited its use in emergency departments. Owing to the misuse and abuse of opioids in some countries worldwide, ketamine has become a versatile tool for sedation and analgesia. In this narrative review, ketamine’s role as an analgesic is discussed, with both known and new applications in various contexts (acute, chronic, and neuropathic pain), along with its strengths and weaknesses, especially in terms of psychomimetic, cardiovascular, and hepatic effects. Moreover, new scientific evidence has been reviewed on the use of additional drugs with ketamine, such as magnesium infusion for improving analgesia and clonidine for treating psychomimetic symptoms. Finally, this narrative review was refined by the experience of the Pain Group of the Italian Society of Emergency Medicine (SIMEU) in treating acute and chronic pain with acute manifestations in Italian Emergency Departments.

## 1. Introduction

Since its introduction in the mid-1960s, ketamine has been a controversial topic. Initially, there was great enthusiasm about its potential uses. However, fears arose that hindered distribution and limited availability. Nevertheless, interest in ketamine has recently increased, especially in emergency medicine, psychiatry, and other professions [1]. Ketamine rapidly induces general anesthesia while preserving the patient’s protective reflexes and vital functions. However, the benefits of ketamine have somewhat diminished over time due to concerns about its psychomimetic effects [1,2]. This drug was originally developed by Craig Newlands and later synthesized by Calvin Stevens in 1962 [3,4]. The aim was to develop a safer and more manageable drug than phencyclidine (PCP), from which it was derived [5,6]. Soon after its development, the analgesic effect of ketamine was discovered. Research in 1965 demonstrated the analgesic effect of ketamine and suggested the use of subdissociative doses of ketamine during painful procedures in children [5,7]. In 1971, the analgesic effect of ketamine was further confirmed when it was observed that patients who underwent anesthesia with ketamine required less opioid medication and experienced better pain management [8,9]. By the mid-1970s, it became clear that different doses of ketamine had different effects [10]. A sub-dissociative dose of 0.6 mg/kg was identified as effective for analgesia, although fixed analgesic doses are still used [11]. The ability of ketamine to produce different effects depending on the dosage is unique among drugs [11]. However, concerns about its side effects have led to a gradual decline in its use [12]. Nevertheless, ketamine has recently experienced a resurgence in emergency departments, and its use is increasing rapidly. It is now used as a sedative, analgesic, antidepressant, and emergency rescue medication for super-refractory status epilepticus [1,13,14]. Due to its profile, ketamine is now considered one of the most important drugs for sedation and analgesia in critically ill patients [15] with haemodynamic instability [16,17].

The most used form of ketamine consists of a racemic mixture of two enantiomers, S-ketamine and R-ketamine, which contain an asymmetric carbon atom [6,18]. The two molecules have different antidepressant, psychomimetic, and analgesic effects [11,19,20,21]. However, this review focuses on racemic mixtures, as data on the specific use of either enantiomer have not been fully established [22]. The analgesic effects of low-dose ketamine are diverse, and its spread is partly due to the opioid crisis in the United States [23,24,25]. Low-dose ketamine has been shown to have an opioid-sparing effect and has been shown to reduce opioid tolerance [24]. Ketamine plays an important role in chronic pain in which the spinal cord is decompressed. In addition to its role as an analgesic in acute pain [26,27], ketamine can reduce hyperalgesia and allodynia in chronic pain [28,29]. Furthermore, there is increasing evidence of its antidepressant effect, which is particularly important in patients with chronic pain [30]. The analgesic role of ketamine metabolites has not been clearly established [18,31,32,33,34,35], but their antidepressant effects seem to be more evident [36]. Ketamine is essential when opioid analgesia is contraindicated or problematic [26], e.g., in elderly patients, patients with OSAS or COPD, patients on chronic opioid therapy, and patients with drug dependence or a history of drug dependence [37]. It is important to emphasise that pain is one of the most common reasons for emergency department visits [38]. The high safety profile of ketamine compared to the many adverse effects of opioids [39] suggests that prescription strategies for acute pain need to be redefined [40].

The objective of this narrative review was to explore the expanding role of ketamine as a unique analgesic drug to define the benefits and possible risks of a therapy gaining great interest [2]. This analysis addresses established and expanding indications for ketamine based on currently available evidence, focusing on areas of uncertainty and potential future developments. Therefore, a review of ketamine’s analgesic role is essential to provide a basis for further studies, as there are no established standards for dosing and administration [41]. An important aspect of the review is the investigation of adverse events, which are sometimes complicated by reports of neurological, bladder, or psychological damage in patients abusing ketamine as a substance. In addition, some studies frequently report various psychomimetic effects of ketamine at sub-dissociative doses, which presents a challenge in standardizing adverse events [42]. Finally, we referred to the experience of the Sedation and Analgesia in Urgency (SAU) group of the Italian Society of Emergency-Urgency Medicine (SIMEU) with the use of low-dose ketamine, which gives an insight into the situation in Italy [43,44].

## 2. Materials and Methods

To conduct this narrative review, we searched for articles published in English on PubMed Medline (last search 31 January 2023) without any time constraints on the pharmacokinetics, pharmacodynamics, and clinical applications of ketamine. Our search fields included keywords such as “ketamine”, “low dose ketamine”, “sub-dissociative ketamine”, “analgesia”, “pharmacokinetics”, “pharmacodynamics”, “neuropathic pain”, “chronic pain”, “cancer pain”, “opioid-sparing effect”, “opioid tolerance”, “OIH”, “headache”, “adverse effect”, “acute pain”, “infusional”, “antidepressant”, “abuse”, “topical anaesthetic”, and “intranasal”. This review addresses: (i) drug characteristics with emphasis on the antidepressant effect of ketamine and its effect on chronic pain, the interaction between ketamine and magnesium, the relationship between ketamine and the opioid system, and its anti-inflammatory properties; (ii) clinical use of ketamine with emphasis on postoperative pain, chronic and neuropathic pain, cancer-related pain, headache, and the use of ketamine as a local anesthetic; (iii) adverse events and their management; (iv) monitoring of patients treated with low-dose ketamine; (v) risk of abuse; and (vi) contraindications. Studies that addressed the sedative, dissociative, and antidepressant effects of ketamine were excluded.

## 3. Drug Characteristics

− Pharmacokinetics

Ketamine is a highly lipophilic molecule with rapid distribution and immediate passage through the central nervous system. It has low plasma protein binding, ranging from 10% to 50%, an alpha half-life of 2–4 min and a beta half-life of 2–4 h [1,6,44]. Owing to its high liposolubility, it has a large volume of distribution, ranging from 160 to 550 litres [6]. The liver metabolizes ketamine via the cytochromes CYP 2B6 and CYP3A4, producing (R, S)-norketamine, which is converted to 6-hydroxynorketamine and 5,6-dehydronorketamine [45]. These metabolites have an extended half-life of up to 3 days and, according to various authors, provide prolonged analgesic and antidepressant effects, which will be discussed later [32,36,45,46]. Bioavailability and duration of action vary depending on the route of administration: with intravenous administration, bioavailability is 100% and maximum effect is achieved within 1–2 min [39,45,47]; with intramuscular administration, bioavailability is 93% and maximum effect is achieved within 5–10 min [39,45,47]; with oral administration, bioavailability is 16–29% and maximum effect is achieved within 20–120 min [45]. Intranasal administration shows a bioavailability of 35–50% [48,49,50], an analgesic effect with onset of action within 10 min, a time-to-peak effect of 10–14 min [51] and a duration of up to 60 min [52]. Oral administration of ketamine is considered less beneficial because of its lower bioavailability and significant hepatic first-pass effect [6,18]. As ketamine is one of the most commonly abused psychoactive substances worldwide, its oral route is not recommended because of its potential for abuse. The intranasal route of administration has been discussed in a separate section. Ketamine and its metabolites are excreted from the body mainly by the kidneys [45]. Women generally metabolize ketamine more rapidly (up to 20%) than men, whereas older people tend to metabolize it more slowly [45]. Ketamine is contraindicated during pregnancy and lactation [11,24]. Due to its short half-life, no dosage adjustment is required in patients with impaired renal function [29].

Owing to its pharmacokinetic properties, the intravenous route of administration is the most beneficial for acute pain. Ketamine has a faster onset of action than morphine but a shorter half-life [53].

− Mechanism of action

The main mechanism of action of ketamine is to block glutamatergic neurons via its antagonistic effect on NMDA receptors [1,44]. It does this by non-competitively blocking the opening of glutamatergic channels, mainly in the prefrontal cortex and hippocampus [4,45]. Ketamine also activates the prefrontal cortex via blockade of inhibitory interneurons, which is one of the mechanisms responsible for its psychomimetic effects [3]. The effect of ketamine on NMDA receptors is unique in that it acts as an open-channel blocker. It blocks the calcium channel only when it is open and has no effect on the closed resting channel [47]. However, the analgesic effects of ketamine are diverse and multifaceted, with effects on dopaminergic [54,55,56], adrenergic [57,58], serotoninergic [59], opioid [58,60] and cholinergic [52,61] receptors. Ketamine acts on the latter by stimulating the nicotinic pathway and inhibiting the muscarinic pathway by blocking M1 receptors, which explains the mydriasis and sialorrhea observed at dissociative doses [52,61]. In addition, ketamine acts on spinal GABA interneurons [62].

Ketamine modulates the reuptake of serotonin, dopamine, and norepinephrine and causes a paradoxical increase in glutamate with stimulation of the descending inhibitory pathways [45,56]. This broad and diverse spectrum of action is essential for its antidepressant effect, which is of particular interest for chronic and neuropathic pain [1]. The blockade of NMDA receptors by ketamine is involved in reducing spinal cord exhaustion, which is a major contributor to the development of chronic pain [4]. Severe pain activates NMDA receptors with hyperexcitability of spinal interneurons in the posterior horn, leading to spinal cord wind-up and central sensitisation [63]. The paradoxical increase in glutamate is essential for the stimulation of medullary GABA inhibitors [45] and for the stimulation of AMPA receptors, which are crucial for the control of depressive symptoms [45,64,65]. Ketamine blocks the NMDA-Rs of GABAergic interneurons, leading to a paradoxical increase in extracellular glutamate and activation of the α-amino-3-hydroxy-5-methyl-4-isoxazolepropionic acid receptor (AMPAr), which stimulates the mammalian target of rapamycin complex-1 (mTORC1) signalling pathway, particularly in cortical excitatory pyramidal neurons [66]. AMPA blockade inhibits the antidepressant effect of ketamine [67,68]. Ketamine also has an anti-inflammatory effect by lowering the levels of IL-6 and TNF-alpha [69]. Increased levels of dopamine in the prefrontal, frontal, nucleus striatum, and nucleus accumbens are other causes of psychomimetic symptoms triggered by ketamine, which can mimic schizophrenic symptoms [70,71]. Serotonin also plays an important role in pain modulation, and blockade of 5-HT2 receptors in mouse models reduces the analgesic effect of ketamine [72].

In the past, great importance was placed on the analgesic role of ketamine metabolites, but this has since been revised [36]. However, experimental evidence in animal models suggests that norketamine plays an essential role in hyperpolarizing the HCN channels in the spinal cord and hippocampus, which is particularly important for antidepressant modulation by ketamine [45,46]. However, in animal models, HCN receptors appear to be involved in the analgesic effects [73,74,75,76]. The action of ketamine on opioid receptors does not appear to have a direct analgesic effect but does have a modulatory effect. Direct intrathecal antagonism of mu and delta receptors (but not kappa receptors) blocks the analgesic effect of ketamine, which is not affected by the parenteral administration of naloxone [77]. The effects of ketamine are extensive and are still largely misunderstood. It acts on sigma-1 receptors, L-type voltage-gated calcium channels, and voltage-gated sodium channels, but their exact functions and possible roles in analgesia are not yet known [1,4].

Ketamine reduces the reuptake of catecholamines at the neuronal level, resulting in increased levels of norepinephrine, dopamine, and serotonin, thus increasing the catecholaminergic tone [6]. However, the effects of ketamine on the cardiovascular system remain unclear. For example, ketamine has a negative inotropic effect only in patients with catecholamine deficiency due to direct myocyte blockade, as seen in patients with severe trauma or intensive care [4]. The neuronal interaction of ketamine is even more complex; while ketamine has raised concerns about neuronal damage in animal models [78,79], there is evidence of a neuroprotective effect of ketamine in the presence of acute stress [80,81,82,83]. Indeed, ketamine has been shown to protect against hyperammonemia-induced lethality in acute portosystemic encephalopathy through nitric oxide- and glutamate-mediated neuroprotection, with reduced neuronal oedema [84,85,86,87,88,89]. A similar neuroprotective effect was observed in patients with super-refractory epilepsy [14]. The exact mechanism by which ketamine exerts its neuroprotective effects is not yet fully understood. However, it has been suggested to increase neuronal calcium while inhibiting calmodulin activation, NO synthase, and NO production from L-arginine, resulting in a neuroprotective effect [90].

Ketamine has a broad spectrum of actions and undeniable benefits for the protection of respiratory function. Unlike opiates, ketamine does not induce respiratory depression [1]. In addition, unlike natural opiates such as morphine, which can cause bronchosthenosis in asthmatics [91], ketamine acts as a bronchodilator [18,92,93].

− Ketamine’s antidepressant action and its effect on chronic pain

This review will not go into detail about the antidepressant effects of ketamine, but there is an interesting link between pain and depression: functional and neuroimaging studies have shown that ketamine reduces the activity of the insular cortex and thalamus, which are normally activated by pain [11,94,95]. Although the effect of ketamine on NMDAR receptors has not been fully elucidated, some observations have suggested that these receptors play a crucial role in the context of depression and chronic pain. Specifically, ketamine increases neuronal calcium via NMDAr blockade, which causes a secondary decrease in NMDA-R receptors via gene depression, thereby increasing levels of brain-derived neurotrophic factor (BDNF), which are low in mouse models of induced depression and whose levels are increased by ketamine [96]. In addition, ketamine has been shown to decrease receptor affinity for substance P, a neurotransmitter that increases in chronic pain and is one of the mechanisms underlying the loss of medullary pain inhibition [97,98]. In addition, ketamine appears to block acetylcholine muscarinic receptors (m1ChRs), which may also play a role in modulating chronic pain. Studies suggest that agonists of these receptors may increase the pain threshold [61,99]. In addition, animal studies have suggested that ketamine may modulate astrocytic and glial responses that play a role in chronic neuropathic pain [100,101].

As mentioned in the Introduction, the exact analgesic effect of ketamine metabolites is not yet fully understood. However, some studies suggest that these metabolites have analgesic properties equivalent to one-third of the analgesic effect of ketamine [33,34] and tend to accumulate over longer infusion periods, resulting in a sustained analgesic effect over several days [102,103].

The relationship between ketamine, serine, depression and chronic pain is of great interest because NMDA-Rs require D-serine or glycine as co-agonists, especially in neurons in the limbic region involved in the development of depression and chronic pain. Serine racemase produces D-serine in medullary interneurons, and its level increases during neuropathic pain, leading to the activation of NO synthase. Ketamine may also interfere with this level [104,105].

However, it is worth noting that depression and chronic pain are closely linked [106], and a drug that can effectively treat both conditions would be ideal [23,107,108,109]. Indeed, ketamine appears to have a stronger analgesic effect in patients with chronic pain and depression [110]. The first observation of the antidepressant effects of ketamine dates to the 1970s. However, it took many years to gain acceptance in this field [45], and the first major study on this topic was conducted in 2000 [111].

The mechanisms underlying the effects of ketamine are not fully understood. However, there is evidence of a synergistic effect between ketamine and lithium as antidepressants [112]. The paradoxical increase in extraneuronal glutamate by ketamine appears to disinhibit pyramidal neurons, activate AMPAs and TORC1s, increase levels of GABA-B and BdNF, and inhibit glycogen synthase kinase 3 (GSK-3B) in the brain, and lithium also affects GSK-3B. As a result, these two drugs can act synergistically to produce enhanced antidepressant effects [112].

− Synergistic effects of ketamine and magnesium

The first observation of the analgesic effects of magnesium and ketamine dates to 1971 [113], and the relationship between magnesium and ketamine is intriguing. Magnesium binds to the NMDAR receptor channel at rest, whereas it does not bind when the channel is active [96]. Magnesium is the body’s NMDA receptor antagonist, and the binding site of ketamine to the same receptor is nearby [96]. The presence of magnesium increases the binding affinity of ketamine for NMDA receptors, and their association with analgesic purposes becomes even more interesting when one considers that brain magnesium levels are reduced in both depression and chronic pain [114]. The addition of 50 mg/kg magnesium to the analgesic ketamine bolus and 10 mg/kg/h magnesium to the ketamine infusion seems to enhance the analgesic efficacy of ketamine [115,116,117,118,119,120,121]. However, it is not yet known how long magnesium infusions should be administered. Magnesium also appears to improve haemodynamic stability [122].

− Relationship between ketamine and the opioid system

As mentioned earlier, ketamine has a complex relationship with opioid receptors. By interacting with central and spinal opioid receptors and NMDA-R, it reduces opioid tolerance, opioid-induced hyperalgesia (OIH) and central sensitisation [6,24,63,123,124,125]. Although opiates reduce pain perception by activating mu receptors, they also activate NMDA receptors, leading to postsynaptic hyperexcitability, central tolerance, and sensitization. Ketamine has been shown to modulate and reduce these effects when combined with an NMDA antagonist such as MK-801 [3,126,127,128,129]. In addition, ketamine exerts a downstream effect by increasing opioid-induced phosphorylation of extracellular signal-regulated 1/2 kinase (ERK 1–2), so fewer opioids are required to achieve the desired therapeutic effect (opioid-sparing effect). This also helps reduce adverse events such as respiratory depression and vomiting [39,58,130,131,132,133,134,135].

− Ketamine and its anti-inflammatory properties

Pain is an important inflammatory component, particularly in the postoperative setting [45]. Ketamine decreases IL-6, TNF alpha, CRP, and NO synthase levels. High levels of IL-6 are associated with poor postoperative outcomes [69]. Chronic postoperative pain can occur in up to 20% of all surgeries and 50–60% of surgeries involving nerve structures [136]. Postoperative neuropathic pain is caused by activated microglia in the spinal cord [3,69,136,137,138]. Therefore, the effect of ketamine on glial cells [100,101] can alleviate postoperative pain.

## 4. Clinical Settings

Low-dose ketamine has numerous applications in treating pain, including acute pain in the emergency department and postoperative, chronic, and neuropathic pain [1]. However, the use of ketamine for headaches and neoplastic pain is not well defined [1]. Ketamine is indicated for treating severe pain, but no benefit has been observed for moderate or mild pain [139]. Evidence for the efficacy of low-dose ketamine remains controversial, with studies providing mixed results [1,2,3,4]. Nevertheless, an increasing number of observations of specific receptor effects support the efficacy of ketamine. For example, PET studies have shown high ketamine binding in brain regions with high NMDA receptor density that are directly involved in pain modulation, such as the somatosensory cortex, insula, and anterior cingulate cortex [2,95,140]. The optimal analgesic dosage of ketamine varies widely in the literature, ranging from 0.15 to 0.5 mg/kg [45]. However, doses above 0.3 mg/kg can lead to psychomimetic symptoms, and 0.5 mg/kg is considered a subdissociative dose and is associated with a higher rate of adverse events [28,141,142,143]. As a result, many authors define safe and effective analgesic dosing as 0.15–0.3 mg/kg bolus, 0.15–0.3 mg/kg/h continuous infusion, 0.5–1 mg/kg intramuscular administration, and 1 mg/kg intranasal administration (see Table 1 [45]).

As described in the section on pharmacokinetics, oral administration of ketamine is not standardized, but doses of 0.5 mg/kg every 12 h are considered effective [144]. Other possible routes of administration include transdermal (25 mg/24 h), subcutaneous (0.05–0.15 mg/kg/h), and rectal (10 mg/kg) (Table 1) [145]. As the use of ketamine as an analgesic has increased, it is important to define its precise indications. While many meta-analyses and studies have shown that ketamine has a significant response rate comparable to that of opioids [146], comparison of studies and meta-analyses can be difficult due to differences in dosing, clinical setting, the presence or absence of other concomitant analgesic therapies, and difficulties in assessing and identifying chronic pain due to a lack of standardization [4].

The administration method can be tailored to the clinical setting.

- isolated, repeatable administration for the treatment of acute pain [53]
- continuous administration as an infusion, typically over less than 100 h, for the treatment of chronic, neuropathic, or certain forms of acute pain [53].

Continuous infusion administration takes advantage of the increased levels of ketamine metabolites [33,34,102,145,146] and their analgesic and antidepressant properties [45,46]. Although some hypothesise that the response to ketamine decreases with continuous infusions due to tachyphylaxis, there is no strong clinical evidence to support this [147]. Longer infusions (up to 100 h) resulted in a sustained analgesic response of 4 to 8 weeks, while infusions of 12 to 24 h resulted in a reduced but stable response of 7 to 10 days [41].

Intranasal administration at a dose of 1 mg/kg [52,148,149,150,151,152,153,154,155] has been shown to have few and limited side effects [156,157,158,159,160], and its efficacy and safety are comparable to intravenous ketamine therapy. Maximum blood concentration can be measured within 2 min of administration, with a peak at 30 min [159]. However, a possible side effect of intranasal administration is an unpleasant taste [160].

Treatment of acute pain with low-dose ketamine in the emergency department is perhaps the most well-tested indication [161,162,163]. However, meta-analyses have shown significant differences in adverse events when inconsistent and nearly subdissociated doses (0.5 to 0.6 mg/kg) are used [164,165,166], and comparison with other drugs with similar analgesic effects also makes it difficult to draw conclusions [167,168]. Ketamine appears to be an ideal analgesic in the prehospital setting because of its safety profile and the ability to administer it intranasally [169]. However, given the wide range of health profiles in prehospital emergency services, it is difficult to develop a standard of competence and authorisation for the administration of ketamine [170]. In patients with severe trauma, ketamine seems to be the better analgesic as it improves haemodynamic stability [171,172]. It is also useful when opioids are not an option due to hypotension [173,174,175], and it can be used safely in head injuries as it does not increase intracranial pressure [176,177,178,179,180,181,182]. Several studies in the emergency setting have shown that low-dose ketamine is non-inferior to morphine, fentanyl and other opioids [26,38,60,162,183]. The incidence of adverse reactions to ketamine administration varies according to dose and route of administration, but none of the adverse reactions have altered the patient’s course or led to discontinuation of treatment with ketamine [184]. Administration as a bolus is common in the emergency department, but continuous infusion is also possible and beneficial in some situations [185]. Treatment of acute pain in the emergency department must be rapid and effective, improve haemodynamic stability and reduce the risk of chronic pain [186]. Ketamine in analgesic doses appears to meet these criteria [6,141,164,164,165,166,187] and is extremely useful in patients with opioid dependence and tolerance, OIH or buprenorphine, methadone or naltrexone therapy [26] and in patients with exacerbated neuropathic pain [40]. Ketamine is also used to treat severe acute pain in patients with renal dysfunction and critically ill patients with hypoxic or hypercapnic respiratory failure [26,188].

− Post-operative pain

The benefit of ketamine in reducing inflammation and IL-6 has not been demonstrated in clinical practice [189,190,191], although some authors have indirectly demonstrated its efficacy by reducing opioid use in the postoperative setting [140,192,193,194,195,196,197]. Due to differences in the surgical setting, procedures, timing of ketamine administration, and dosages, meta-analyses are difficult to compare, and a large study with 8000 participants has not yet been completed [198]. Nonetheless, ketamine’s efficacy as an analgesic for acute pain suggests that it can be used as an alternative to or in addition to opioids while reducing their adverse effects (sedation, respiratory depression, and vomiting). Furthermore, the potential benefits in postoperative management may be an additional factor, particularly in major surgery and especially when the nervous system is involved [64,134,198,199,200].

− Chronic and neuropathic pain

The use of ketamine in analgesic doses for the treatment of chronic pain is of interest due to its previously highlighted effects on NMDA-R, the spinal cord, and the glia [3,63,100,101,201,202,203]. Although there is relatively little evidence for it at present, its potential use is very interesting [47]. Chronic pain not only causes severe disability but also imposes significant costs on society, which continue to rise [104,204,205]. The literature on chronic pain is complex due to varying definitions and a lack of standardisation, and the analysis of low-dose ketamine is no exception [34,206,207]. Analgesic-dose ketamine is used in a variety of clinical situations, but the most studied are post-herpetic neuralgia, complex regional pain syndrome type 1 (CRPS-1), fibromyalgia, and axonal neuropathies [103,208,209,210]. However, the strongest indications for its use seem to be chronic neuropathic pain that does not respond to other treatments [205,206,207], such as low back pain with chronic radiculopathy [90,211,212]. On the other hand, its use in trigeminal neuralgia is relatively poorly studied [213].

Chronic pain of any kind is maintained in part by events that ketamine counteracts [138]. These events include hyperactivity and disruption of NMDA-R neurons with central sensitisation [201,214,215,216], loss of descending inhibitory function of interneurons in the spinal cord [217,218], and activation of inflammatory cytokines in the spinal cord with central sensitisation [192,219].

Chronic pain impairs the inhibitory function of the spinal cord, and MRI and electrophysiological studies show that ketamine can help restore this function [140,187]. The modulation of chronic pain by ketamine occurs not only at the level of NMDA-R but also in other pathways that are not yet fully understood [220,221]. Given the close relationship between chronic pain and depression [17,222,223], the antidepressant effect of ketamine appears to play an important role in improving the quality of life of patients with chronic pain [34]. The link between chronic pain and a high risk of suicide should not be forgotten [223,224], and ketamine may modify these risks [90]. Speaking of quality of life, studies do not usually consider the effects (positive or negative) of analgesic therapy on various aspects of a patient’s life, such as driving ability, relationships, and depressed mood [41], which is improved by ketamine thanks to its antidepressant effect [90,200]. Studies suggest that ketamine may be effective in relieving chronic postoperative pain [225], although the best evidence has been observed for the treatment of complex regional pain syndrome [203,226]. Therefore, low-dose ketamine is recommended for complex chronic pain situations as there are few relative contraindications or risks associated with its use [227]. However, it is unclear which patients or types of pain benefit most from an analgesic dose of ketamine [228,229]. Studies suggest that a continuous infusion of ketamine over a longer period is associated with better pain control, even days after administration [103,202,203].

− Cancer-related pain

Ketamine may be a viable option for the management of neoplastic pain in patients who cannot tolerate opioids or have opioid-induced hyperalgesia, resistant pain, or neuropathic pain components [230,231]. However, patients with neoplasms have unique clinical features, and the evidence for the use of ketamine in neoplastic pain is limited [47,232]. Nevertheless, the initial findings are promising [100] and deserve further research [233]. Undoubtedly, psychomimetic symptoms can be a major problem for these patients [234]. In response to the opioid crisis, ketamine has been successfully used in analgesic doses in cancer patients and in palliative care in several countries [234,235]. However, finding the right dosage for the individual patient can be challenging, as higher dosages are associated with stronger psychomimetic effects [232,233,234,235,236]. Unlike opioids, where the dosage is increased gradually to achieve the desired effect, ketamine dosage should remain constant to avoid changes in clinical effect [11]. Neoplastic pain is also difficult to manage, and a distinction must be made between palliative treatment and the treatment of active cancer [237]. Intravenous administration is preferred as subcutaneous administration is ineffective [238]. Similar to chronic pain, the antidepressant effect of ketamine is thought to be a key element in its analgesic effect, which may help to control or reduce the disabling symptoms of cancer patients or palliative care patients [239].

− Headache

Because of the various clinical pictures [240] and the possible forms of drug abuse [241], the treatment of headaches is particularly complex. Nevertheless, ketamine appears to be effective in controlling migraine [242], although the evidence is not particularly convincing [243]. It is important to note that the timing of migraine treatment is critical, as pathophysiology and therapies vary between the prodromal phase with aura (vasospasm) and the headache phase (vasodilation), where ketamine shows good efficacy [244]. While more studies are needed to fully understand the use of ketamine in analgesic dosing for the treatment of headaches, the initial results are interesting because they offer an additional therapeutic option [245,246].

− Ketamine as a local anaesthetic

The idea of using locally administered ketamine to block nerve conduction was proposed as early as 1975, similar to other clinical applications [247]. Later, the direct action of ketamine on sodium channels at peripheral nerve terminals was theorised [248]. Although ketamine does not play a significant role in pain management, the idea of using ketamine to induce local anaesthesia is intriguing and deserves further investigation [249,250,251,252].

## 5. Adverse Events

Analysis of the literature on the adverse effects of low-dose ketamine is challenging, as most of the reported incidents are due to higher doses used for sedation during procedures or to the persistent abuse of ketamine as an illicit substance [25,26,53]. In general, current evidence suggests that ketamine in analgesic doses has a better safety profile than opioids [26,38,253,254]. Psychomimetic effects occur with the use of ketamine in analgesic doses. They are generally dose-dependent and are most observed at doses above 0.35 mg/kg [3,45] (especially at doses between 0.4 and 0.7 mg/kg) [24,47]. These effects include depersonalisation, conceptual and mental disorganisation, hallucinations, and cognitive or emotional blunting [255,256]. They usually disappear within 30 min after the first dose, especially at high doses, or after the start of a continuous infusion [3]. Longer infusions are less likely to produce these effects [202]. While some studies use high doses (0.5 mg/kg), which already have psychomimetic effects [255], other studies show worsening of schizophrenia symptoms even at doses as low as 0.12 mg/kg [256], which is because of ketamine on prefrontal dopamine receptors [70,71], similar to PCP [47]. However, the contraindication to low-dose ketamine in schizophrenia should be put into perspective, as deterioration of the condition usually resolves within 90 min after the end of ketamine administration or infusion [256]. Interestingly, the psychomimetic effects are less frequent and more intense in individuals with depression who have elevated NMDA-Rs [100,257]. Ketamine may also affect memory, but this is based on abuse studies in which the effect of the drug cannot be distinguished from other confounding factors. This effect does not apply to the use of ketamine in analgesic doses, especially when administered acutely or in isolation, but must be considered in prolonged therapy [45]. Analgesic doses of ketamine are associated with several other side effects, including Ketamine may also influence memory, but this is based on abuse studies where the effect of the drug cannot be distinguished from other confounding factors. This effect does not apply to the use of ketamine in analgesic doses, especially when administered acutely or in isolation, but must be considered in longer-term therapy [45]. Analgesic doses of ketamine are associated with several other side effects, including nystagmus and dizziness, which are among the most common side effects that resolve on their own [45]. Tachycardia and blood pressure elevations are uncommon with analgesic doses and usually resolve on their own within 30 min of administration [3,45]. Diplopia and mydriasis are possible but usually minor and resolve spontaneously, even with prolonged infusion [45]. Vomiting occurs less frequently with ketamine than with opioids [26,53,175], but is possible, especially with intramuscular administration [13,258]. Sialorrhoea is more common in young people when ketamine is administered in dissociative doses. It is uncommon at analgesic doses and can be treated with atropine (0–01 mg/kg) [44]. Although transaminase elevations have never been observed with isolated administration, even in patients with severe hepatopathy [34], they do occur with prolonged infusions of more than 100 h duration [23,39,259,260]. However, normalisation of transaminase levels after discontinuation of therapy is also observed with longer infusions [259].

Oral administration of ketamine is associated with a higher rate of adverse events, particularly psychomimetic effects, especially at the beginning of therapy [261]. This is because initial bioavailability cannot be predicted from patient to patient and thus the effective and safe dose cannot be determined in advance. On the other hand, subcutaneous administration can cause irritation and pain [237].

Deaths directly attributable to the abuse of ketamine are very rare (if traumatic causes are excluded) because the therapeutic range of ketamine is very wide. In rats, the lethal dose of ketamine is well above 40 mg/kg [45], while in humans it is about 60 mg/kg, or 4.2 g in 70 kg subjects [42]. In fact, no medical deaths from ketamine are known in the literature, and non-fatal cases have been reported with the administration of up to 10 times the maximum analgesic dose [4]. Even in paediatric cases treated with an overdose of 5–100 times the therapeutic dissociation dose, no apparent harm occurred, apart from protracted dissociation [257,262].

Other effects reported with chronic abuse do not suggest analgesic use of ketamine, such as erosive cystitis [6,45,47,263,264] or memory impairment and decreased cognitive abilities [4,6].

One of the main concerns limiting the therapeutic use of ketamine is the possibility of neurotoxicity. Olney lesions, i.e., cytoplasmic vacuolar lesions observed in brainstem neurons of rats treated with NMDA-R antagonists such as ketamine, phencyclidine and MK801, have been observed with continuous high-dose infusions above 20 mg/kg [78,79]. However, these lesions regress within 24 h after discontinuation of the infusion [45]. On the other hand, ketamine has been found to have a neuroprotective effect in acute conditions such as head injury [81,82,181], where neuronal damage is mediated by an increase in glutamate, which stimulates NMDA-Rs, leading to a further increase in glutamate [11,265], and ketamine can interrupt this vicious cycle. Ketamine can interrupt this vicious cycle. Ketamine also reduces TNF-alpha-mediated damage in the hippocampus, as occurs in acute inflammation [83,266]. We have already mentioned the neuroprotective role of ketamine in super-refractory status epilepticus [14] and acute portosystemic encephalopathy [84,85,86,87,88,89], but it also seems to exert a neuroprotective effect in ischaemic stroke [267].

In summary, neither animal models nor illicit drug abuse data on ketamine can be used to automatically assess the adverse effects of ketamine when used in analgesic doses. The reason for this is that in the first case, the models and doses are neither consistent nor fully transferable to humans. In addition, numerous confounding factors must be considered when analysing data on ketamine abuse [4,40,268,269]. Meta-analyses are also limited because dosing and definitions of adverse events are not consistent, making it difficult to draw definitive conclusions [270].

## 6. Management of Adverse Events of Low-Dose Ketamine

Adverse events associated with low-dose ketamine include psychomimetic symptoms, which may be mild or moderate and can be well controlled by benzodiazepines such as diazepam, bromazepam or midazolam [271,272]. These drugs are well tolerated and have a low risk of other adverse events [271,272]. Clonidine, a central alpha-2-adrenergic agonist with sedative effects, has been hypothesised to counteract the central and peripheral dopaminergic stimulus of ketamine and reduce both psychomimetic and vascular symptoms [34,38,202]. The combination of ketamine with dexmedetomidine is also promising [273,274,275,276]. Preparing the patient for possible psychomimetic side effects and managing them in a calm environment can reduce the magnitude of these effects and transform them from unpleasant to acceptable or even pleasant [34]. Inadequate preparation and use of doses greater than 0.35 mg/kg may result in unpleasant dreams for up to 3 days after administration [277]. A thorough knowledge of the specifics of ketamine is therefore essential, as the experience and knowledge of those administering it can reduce the magnitude of adverse events, especially psychomimetic ones [278,279]. Vomiting can be adequately controlled with ondansetron [38].

## 7. Monitoring the Patient Treated with Low-Dose Ketamine

Ketamine is a dissociative dose that requires continuous monitoring. However, there is no established standard for analgesic dosing. ECG monitoring before and, if necessary, during administration is recommended, as is blood pressure monitoring before and, if necessary, at intervals during treatment. There is currently no evidence for continuous digital saturation monitoring [17].

## 8. Risk of Abuse

The risk of abuse of ketamine in analgesic doses is generally considered to be low [279,280,281], although the exact risk is not known [282]. Although ketamine is one of the most popular substances of abuse [4,283], it is impossible to infer risk from the literature in this area because of the variation in doses and routes of administration. In addition, data on plasma concentrations in people who abuse ketamine recreationally are lacking [45]. Animal models suggest that continuous administration of ketamine leads to tolerance and dependence [47]. In humans, it leads to tolerance and psychological dependence, but not physical dependence, as discontinuation does not produce withdrawal symptoms [284]. However, given the high social risk, a high threshold of attention to this drug needs to be maintained and its use controlled, especially about the psychological dependence maintained by dopamine [42,100,285].

It is important to note that ketamine is also used to treat and control other addictions. Due to its effect on NDMA-Rs, it can delay the onset of alcohol and heroin withdrawal syndromes, reduce cocaine addiction, and promote neuroplasticity by remodeling pathological neural circuits [286]. Ketamine also blocks and modifies pathologically reinforcing mnemonic circuits and exerts significant antidepressant effects [286]. In addition, ketamine interacts with the opiate system, reduces tolerance, has opioid-sparing effects, reduces OIH and combats dependence [6,24,63,124,125,126]. Given these properties, the potential of ketamine to induce dependence must be evaluated considering its ability to control other addictions [280,281,287].

## 9. Contraindications

There are some absolute contraindications to the use of ketamine. These include (i) administration within the first three months of life due to possible neuronal damage during development as shown in animal models [202]; (ii) during pregnancy as suggested by research studies [11,24]; (iii) in patients with hepatic porphyria [11]; (iv) and in patients, there are some absolute contraindications to the use of ketamine. These include (i) administration within the first three months of life due to possible neuronal damage during development, as shown in animal models [202]; (ii) during pregnancy, as suggested by research studies [11,24]; (iii) in patients with hepatic porphyria [11]; (iv) and in patients with a known allergy to ketamine [24]. Other contraindications can be derived from the dissociative dosages, but they do not automatically apply to the analgesic dosages [11]. Therefore, these contraindications may be relative rather than absolute [17]. Caution should be exercised in patients with severe and uncontrolled hypertension [3], persistent heart failure [24], psychosis [45] and glaucoma (based on animal models only) [11]. Head trauma is no longer considered a contraindication [179,180,181,182]. However, these contraindications are rarely observed in patients treated with low-dose ketamine [39].

Liver failure must be considered separately. Acute and isolated administration of ketamine does not worsen liver function, even in patients with advanced cirrhosis [34]. However, continuous infusions may cause an increase in transaminases that are self-limiting [282] but require increased caution in patients with cirrhosis [26,39,259,260].

## 10. Conclusions

Ketamine has been rediscovered by emergency medicine [1] for a variety of indications ranging from procedural sedation [5,258] to rapid and safe immobilisation of patients with psychosis or excited delirium [288]. This is partly due to its ability to exert neuroprotective effects in various situations [289]. The antidepressant effects of ketamine [3,22,290], which have been explored in psychiatry, may also be of undeniable benefit in pain management.

Evidence for the efficacy of low-dose ketamine in various clinical settings is accumulating in the literature [2,3,4,200]. However, the large methodological differences make it difficult to compare studies [172]. For acute pain, non-inferiority to opioids is now well established [162], and ketamine is among the safest options for critically ill patients or those with haemodynamic instability [163]. The ability to administer it intranasally makes it very practical in emergency situations [148].

In chronic and neuropathic pain, the main advantage of ketamine in modulating pain is its simultaneous action on different signalling pathways, although these are not yet fully identified or understood. Ketamine may prevent hyperactivity of the opioid system (leading to tolerance, OIH and central sensitisation), exert an opioid-sparing effect, reduce the pain response, counteract hyperalgesia and allodynia, reduce spinal cord wind-up and restore descending inhibitory pathways. Ketamine in continuous infusions plays an important role as an antidepressant, which is essential for the treatment of patients with chronic pain [2,3,4,162,163,164]. Ketamine is also essential in patients with opiate tolerance or dependence, OIH, dependence on other substances, methadone, buprenorphine, and naltrexone therapy [211,231].

For palliative therapies and the treatment of neoplastic pain, the evidence is still limited, as it is for the treatment of headache, but there is evidence of considerable interest [236].

More extensive studies are needed to define the role of ketamine more precisely. However, the Sedo-Analgesia in Urgenza (SAU) group of the Società Italiana Medicina d’Emergenza-Urgenza (SIMEU) has been using ketamine in analgesic doses for many years, and our experience confirms what is reported in the literature. Ketamine is an excellent drug for the treatment of severe pain in acute cases, but also has remarkable benefits in chronic pain infusions, especially in neuropathic pain such as radiculopathy. Unfortunately, there is currently no clinical evidence to predict individual patient responses. Knowledge of the specifics of the drug and appropriate management are critical to improving the management of adverse events and reducing their prevalence. In our experience, adverse events are kept within limits and rarely limit therapy. Training in analgesic therapy with ketamine is essential for proper use. The SIMEU SAU group has trained more than 5000 physicians and nurses in various areas over the past decade, including low-dose analgesic therapy with ketamine [291]. We have carried out surveys that have shown an increase in the use of ketamine in analgesic doses, and retrospective analyses are underway, and prospective observational studies are planned that will serve to define more precisely the fields of application of ketamine in analgesic doses. 

## 11. Future Directions

While the pharmacodynamic properties of the drug and its increasing use in emergency medicine are encouraging, rigorous research is still needed. Identifying clinical factors that can predict a patient’s response to ketamine will help clinicians determine the most appropriate treatment option. Given the opioid crisis, such studies are more urgent than ever. Future research should also investigate ketamine enantiomers and the development of molecules with more targeted analgesic effects and fewer psychomimetic side effects. Nevertheless, all healthcare providers involved in the treatment of acute, chronic, neuropathic, or neoplastic pain need to be aware of this treatment option and be able to manage its unique side effects. Educational campaigns conducted by scientific societies can be an effective means of achieving this goal quickly and efficiently.

## Figures and Tables

**Table 1:** Analgesic doses of ketamine administered via different routes.

LOW DOSE KETAMINE (ANALGESIC)	
Administration Route	Dose
Intravenous	0.15–0.3 mg/kg bolus 0.15–0.3 mg/kg/h infusion
Intramuscolar	0.5–1 mg/kg
Intranasal	1 mg/kg
Oral	0.5 mg/kg every 12 h
Transdermal	25 mg/24 h
Subcutaneous	0.05–0.15 mg/kg/h
Rectal	10 mg/kg

## Data Availability

Not Applicable.
